# Self-efficacy, grit, and rural career aspirations among early career nurses: a repeated cross-sectional study

**DOI:** 10.1186/s12912-024-01723-4

**Published:** 2024-01-18

**Authors:** Daniel Terry, Blake Peck, Ed Baker, David Schmitz

**Affiliations:** 1 School of Nursing and Midwifery, University of Southern Queensland, 4305 Raceview LPO Raceview, QLD PO BOX 4393, Australia; 2 Centre for Health Research, University of Southern Queensland, Toowoomba, QLD Australia; 3 Institute of Health and Wellbeing, Federation University Australia, Victoria, Australia; 4 Center for Health Policy, Boise State University, Boise, ID USA; 5 Department of Family and Community Medicine, University of North Dakota School of Medicine and Health Sciences, Grand Forks, USA

**Keywords:** Student, Career, Grit, Rural, Aspiration

## Abstract

**Background:**

Global nursing workforce shortage represents an impediment to the delivery of safe, evidence-based healthcare. Despite collective efforts, a consistent stream of nurses leaving the profession remains, particularly within the first five years of practice, which is exacerbated in rural communities. The aim of the study was to compare self-efficacy, grit, and rural career aspirations among nursing graduates between their second and fourth year of their nursing profession.

**Methods:**

As part of a longitudinal investigation, a repeated cross-sectional design was utilised. Participants included, 117 (response rate 52.2%) who completed an online questionnaire 18–24 months after graduating, and 32 participants (response rate of 21.0%) who agree to repeat the questionnaire 36–48 months after graduating. The questionnaire included demographic, employment, and measures examining general and occupational self-efficacy, grit, and rural career aspirations.

**Results:**

No differences between general and occupational self-efficacy or grit were identified between second- and fourth-year nurses. In addition, the importance placed on undertaking rural career also remains unchanged. However, a higher proportion of fourth year nurses were more likely to be in management or were considering leaving the profession.

**Conclusions:**

This examination of early career nurses, now in their second and fourth-year post-graduation highlights self-efficacy, grit, and rural career aspirations remains stable between two- and four-years following graduation, while nursing in their fourth year were more likely to consider leaving the profession. Nursing retention is a ‘Wicked Problem’ that is unavoidably a complex amalgam of macro, meso and micro factors that we are yet to fully appreciate.

## Introduction

The international landscape of nursing and the holistic development of junior or Early Career Nurses (ECN), within the first five years after graduation, is of paramount concern. Among those nurses who graduate from undergraduate programs, up to 25% will leave the profession within the first five years, and nurses leaving the profession early is cause for major concern [1, 2]. As such, the challenges associated with the retention of a skilled nursing workforce poses a significant impediment to the delivery of safe and evidence-based healthcare [3]. To address these challenges, there has been considerable human and financial investment into examining what aspects of healthcare organisations and key interventions that positively influence actual or intended turnover of nurses [4, 5].

Despite the volume of work already undertaken, the projected shortfall of nurses globally signals a level of myopia within current policies and is suggestive that something remains missing in our understanding in order to address these challenges [1, 6]. Within this context, the multifaceted nature of nursing demands an examination of the knowledge, skills, and psychological attributes that contribute to staff retention. Among these attributes, the concepts of self-efficacy, grit, and career aspirations, which encompass ambition regarding what type of employment and specifically where that employment occurs, holds significant implications for the success and professional resilience of ECNs [7].

Self-efficacy, rooted in Bandura’s seminal work [8], embodies an individual’s belief in their competence to accomplish specific tasks and achieve desired outcomes. Within the nursing context, self-efficacy transcends proficiency, but encompasses clinical judgment, communication skills, and patient-centred care. Substantive evidence highlights the pivotal role of self-efficacy in shaping ECNs’ professional trajectory, with low levels linked to poor patient outcomes and staff turnover [3]. Nevertheless, the elasticity of self-efficacy offers a promising avenue for its development, as ECNs can harness diverse avenues for its expansion—such as experiential learning, observation of peers, and constructive feedback from mentors [3]. As such, it is vital to examine if levels of occupational (nursing) self-efficacy and general self-efficacy levels change among nurses relevant to time within the profession and associated challenges of the workplace.

Concurrently, grit, a construct pioneered by Duckworth et al. [9], underscores persistence to overcome challenges and a consistency of interest required to achieve long-term goals. This is particularly relevant among ECNs, where a sustained commitment to navigate workplace is required [10]. Grit’s association with academic achievement and performance outcomes further accentuates its role as a success determinant, surpassing traditional markers of aptitude [11–14]. It has been demonstrated that self-efficacy remains a predictor of grit, given self-efficacy is the self-belief and motivation that one can undertake and achieve difficult tasks, cope with challenges, or produce anticipated outcomes [15, 16]. Further, when enabled through greater knowledge, understanding, and experience, self-efficacy has a greater impact or predictability on the persistency of effort for long-term goals. As such, within the sphere of early career nursing, grit assumes particular significance as a catalyst for sustained effort, adaptive learning, and the capacity to thrive amid evolving challenges, however, an understanding regarding how or if grit levels change relevant to time and experience as a nurse remains relatively unknown.

Further, tenants of Bandura’s [17] social cognitive theory, which encompasses the complex interplay between personal, behavioural, and environmental determinants enables the capacity to situate the integrative role of psychological factors such as grit and self-efficacy have in influencing of career aspirations, specifically aspirations of rural careers. Cosgrave et al., [18] has suggested that employment decisions and where employment occurs, particularly among health professionals, is associated with the strong social bonds, familiarity with the physical environment, and sense of enjoyment may be provided through rural lifestyle. However, Cosgrave et al. [19], later argued that life stage, rather than rural origin, was the major determinant employment decision and where employment was chosen to occur, such as undertaking health professional work in rural contexts. As such, key drivers of grit, such as self-efficacy, remain essential among nurses to empowers them as they encounter challenges, contemplate leaving the profession, or consider complex yet fulfilling work environments, such as those offered in rural healthcare employment [20].

Within this context, the Nursing Community Apgar Questionnaire (NCAQ) serves as an evidence-based tool that gauges the significance ECNs attribute to various factors—geographic, economic, management, practice, and support—when considering rural nursing employment [7, 21, 22]. Past exploration sought to uncover how ECN’s perceptions of pursuing a rural nursing career evolve over time, particularly during the transition from student to registered nurse. It was demonstrated that the level of importance students place on pursuing a rural career over time did not change from student to new graduates over a two years period [7]. However, further exploration is required to understand if additional changes, if any, occur relevant to increased time in the nursing profession beyond their initial two years. By discerning these shifts in importance, practitioners, health care agencies, and services more broadly can gain insights to inform future nursing recruitment and retention efforts, enhancing workforce planning and contributing to the broader healthcare landscape.

Within the context of the self-efficacy, grit, and career aspirations, the aim of the study was to re-examine a single cohort of students at the end of the second year in the nursing profession post-graduation, and again at the end of their fourth year in the nursing profession post-graduation and provide comparison. In addition, a secondary aim was to examine if relationships existed between the number of years working as a nurse among ECNs and key factors such as undertaking further study, taking on management roles, and whether they have or were contemplating leaving the nursing profession.

### Hypothesis

1. General and nursing self-efficacy, grit, and importance nurses place on taking up rural practice score would not change between second- and fourth-year nurses (H1); and.
2. Undertaking further study, undertaking management, and contemplating leaving the profession would increase among nurses from their second to their fourth year within the profession (H2).

## Methods

To examine any differences in self-efficacy, grit, rural career aspiration that exist between 18 and 24 and 36–48 months after graduation, a repeated cross-sectional design was used to collect ECN data between 2019 and 2023 in Australia. This is a follow up study to Terry et al. [7], which examined self-efficacy, grit, rural career aspiration among nursing students and graduates, and is couched within a broader longitudinal investigation examining early career nurse career trajectories. Reporting methods adhered to the STROBE guidelines.

### Sample

Participants consisted of all former nursing students (*n* = 224) who graduated from a regional university with rural and regional campuses and were working throughout Australia. These former students who had participated in the initial study indicated they were willing to be contacted biannually and participate in the study after completing their three-year Bachelor of Nursing degree. Of the participants, 117 completed or partially completed the online questionnaire two years after graduating, yielding a response rate of 52.2%. In addition, among those who were in their fourth year after graduation (*n* = 152), 32 completed the same online questionnaire again, yielding a response rate of 21.0% (Table 1). Follow-up emails were sent to former student at each collection point in weeks 2, 4, 6 and 8 post initial invitation to ensure an adequate sample size (*n* ≥ 116) was obtained to meet 95% CI (MOE ± 6%) among second year graduates; and (*n* ≥ 32) was obtained to meet 95% CI (MOE ± 11%) among fourth year graduates.

**Table 1:** Participants by study year and participation

Graduation year	Agreed to participate	Two-year follow up	Four-year follow up
2018	62	28	26
2019	90	33	6
2020	49	44	n/a
2021	23	12	n/a
**Total**	**224**	**117**	**32**

### Data collection tool

Data were collected using a structured online questionnaire, which encompassed a spectrum of demographic variables, including birth year, employment status, income, nursing roles, and engagement in postgraduate education. To ensure continuity, the questionnaire incorporated questions identical to those employed in the initial 2018 data collection phase, so as to detect changes over time [7]. The anticipated completion time for the questionnaire was estimated at 15–25 min. The questionnaire included scales retained from the 2018 phase, which encompassed the following scale items:

- The General Self-Efficacy Scale (GSE-10) where participants self-rate themselves against 10 questions which examine self-efficacy, the general belief in an individual’s ability to respond to difficult situations, obstacles, and setbacks. The scale used a five-point Likert scale (from ‘Exactly true’ to ‘Not at all true’) and demonstrated good reliability of between 0.790 and 0.900 for each individual scale item [23];
- The Nursing Self-Efficacy Scale (NSE-8) developed by Schyns and Von Collani [24] that had 8 occupation specific self-efficacy items. The scale was modified with minor wording changes, where the generic word ‘job’ was replaced with the specific word ‘nursing’. The scale used a five-point Likert scale (from ‘Exactly true’ to ‘Not at all true’) and demonstrated good reliability at α = 0.880.
- The eight-item short grit scale (Grit-S), where participants self-rate against eight items using a five-point Likert scale (Very much like me through to Not like me at all) to measure two distinct constructs, consistency of interest (passion) and perseverance of effort (perseverance) with the reliability of the scale at α = 0.755, [9];
- The Nursing Community Apgar Questionnaire (NCAQ) developed by Prengaman et al. [21], used to measure the level of importance nurses or nursing students place on taking up rural practice. The NCAQ used a four-point forced Likert scale (from ‘Very Important’ to Very Unimportant’), demonstrates good reliability with a Cronbach alpha of 0.961 [21].

### Data collection

Data collection occurred among former nurses working in Australia and within a specified window, spanning from February to May of each year. Participants were extended invitations via email, which provided access to an Information Statement detailing the voluntary nature of participation, potential risks, and a link to the online questionnaire.

### Data analysis

Data analysis was facilitated using the Statistical Package for the Social Sciences (SPSS, Version 28.0). A comprehensive analytical approach was adopted, encompassing both parametric and non-parametric tests. Independent sample t-tests and Mann-Whitney U tests were employed to identify inter-group differences. These tests were selected due to lower than anticipated response rate, which made it impracticable link participant data at data collection points that would have enabled data matching. Additionally, Chi-square tests were used to explore if relationships between years working as a nurse and several variables existed, where effect size (Phi) was considered to have a small (0.1), medium (0.3), and large effect (0.5). Preliminary analyses were undertaken to ensure no violations of assumptions were present and that data distribution adhered to a normal distribution, where acceptable values of skewness and kurtosis fell between ± 3 and ± 10 respectively [25]. Statistical significance was determined at a two-tailed *p* ≤ 0.05, enabling a nuanced exploration of the intricate dynamics underlying nursing career trajectories and factors influencing employment decisions [26].

### Ethical considerations

Ethical approval for the study was procured from the Federation University Australia Human Research Ethics Committee (Approval #18 − 017). All aspects of the research adhered to the ethical principles for medical research on human beings, as set out in the Declaration of Helsinki and all methods were performed in accordance with the relevant guidelines and regulations. Each participant provided informed consent prior to commencing data collection.

## Results

Responses from the questionnaire identified half of the participants (*n* = 64) were aged between 20 and 39 years, with just under half (*n* = 53) living in a large regional centre. Two-thirds (*n* = 83) were working in part-time employment, with a greater proportion of nurses in their fourth year working full-time. Overall, more than half worked in metropolitan or urban centres. (Table 2).

**Table 2:** Participant demographics

Demographic information	Second year Nurse	Fourth year Nurse
	*n*(%)	*n*(%)
Age (years) (*n* = 121)		
- 20–29 years	26(31.7%)	7(17.9%)
- 30–39 years	21(25.6%)	10(25.6%)
- 40–49 years	19(23.2%)	14(35.9%)
- 50 years and over	16(19.5%)	8(20.5%)
Where currently living (*n* = 123)		
- Inner City Metropolitan	3(3.5%)	1(2.6%)
- Outer Suburb Metropolitan	16(18.8%)	6(15.8%)
- Large Regional Centre	36(42.2%)	17(44.7%)
- Small Town/farm	30(35.3%)	14(36.8%)
Employment status (*n* = 124)		
- Working full-time (38hrs/week)	10(11.8%)	9(23.1%)
- Part-time employee (> 38hrs/week)	60(70.6%)	23(59.0%)
- Working Casual employee	12(14.1%)	5(12.8%)
- No in workforce, but looking	2(2.4%)	0(0.0%)
- Not in workforce	0(0.0%)	1(2.6%)
- Not stated	1(1.2%)	1(2.6%)
Location of employment (*n* = 116)		
- Metropolitan/city	31(39.2%)	9(24.3%)
- Urban/Suburban	15(19.0%)	11(29.7%)
- Rural	30(38.0%)	15(40.5%)
- Not stated	3(3.8%)	2(5.4%)
Undertaking study (*n* = 114)		
- Yes	23(28.7%)	11(32.4%)
In management role (*n* = 119)		
- Yes	3(3.7%)	8(21.6%)

When examining occupational or nurse self-efficacy levels among nurses who were in their second- and fourth-year post graduation, they had similar scores. These similar scores were also noted for general self-efficacy, where there was no difference between second- and fourth-year nurses. Beyond self-efficacy among participants, a comparison was made regarding the levels of grit between groups. As such, it was indicated that nurses who were in their second and fourth year of nursing after graduation had no significant difference in grit scores. Lastly, a comparison was made between the level of importance placed on rural career aspirations among nurses. It was shown that there was little variation in scores between the second- and fourth-year nurses (Table 3).

**Table 3:** Comparison of mean scale items

Scale item	Mean (SD)	Test (df) Statistic	*p*
NSE-8 (Second year)	24.37 (2.62)	t(109) =-.887	.382
NSE-8 (Fourth year)	24.84 (2.83)		
GSE-10 (Second year)	31.22 (4.33)	t(110) =-.813	.436
GSE-10 (Fourth year)	31.87 (3.95)		
Grit (Second year)	3.30 (0.41)	t(110) =.336	.738
Grit (Fourth year)	3.27 (0.42)		
NCAQ (Second year)	3.53 (0.41)	t(78) =-.341	.734
NCAQ (Fourth year)	3.63 (0.31)		

In addition, when examining the relationship between the number of years working as a nurse among ECNs and key factors, it was noted a there was no significant difference between groups in terms of undertaking further study (χ = 0.148, df = 1, *p* = 0.700), phi = 0.036. However, a higher proportion of fourth year nurses were more likely to be in management roles than second year nurses (χ = 9.806, df = 1, *p* = 0.02), phi = 0.287. Lastly, there was a higher likelihood among fourth year nurses who were considering leaving the nursing profession compared to second year nurses (χ = 7.127, df = 1, *p* = 0.001), phi = 0.247.

## Discussion

For nurses who are two- and four-years post-graduation there was no difference identified between the two cohorts based on general and occupational self-efficacy or grit measures. Interestingly, there was also no change identified in measures of these variables when this same cohort were final year and newly graduated nurses respectively [7]. Existing literature argues self-efficacy and grit continue to develop throughout professional practice (McCabe, 2016), however, this study indicates these measures are relatively stable between years two- and 4 post-graduation. While previous work has highlighted that first-year post-graduation is a time of growth and development [27, 28], this stabilisation of self-efficacy and grit, seen in this current study, could be construed as a positive transition to a phase of consolidation as a nurse and underpins the relative stability we see among ECNs.

Although grit and self-efficacy have been shown to be relatively stable over time [27], more recent grit was shown to decreased significantly at the height of a major crisis. This suggests the transition for nurses between second and fourth year after graduating, although a time of learning and ongoing development, there is insufficient change or ‘crisis’ to render any change in grit scores [20, 28]. In addition, self-efficacy has been suggested to be further developed among ECNs by harnessing diverse avenues for its expansion, which include experiential learning, observation of peers, and constructive feedback from mentors [3]. However, there was little to no change among nurses relevant to time within the profession and associated challenges of the workplace. These findings may be seen as a positive in that the challenges, growth and developed that are encountered as an ECN is sufficient to no impact their capacity to provide care in challenging environments, however, there has also been little increase in either grit or self-efficacy over this time. We postulate, although ECNs have adequate capacity to persevere and to bounce back, despite challenges within the work environment, there are other elements, not measured here, that may have a greater impact on ECNs contemplating leaving the profession, which requires further examination [4, 5].

In addition, the NCAQ highlights that the importance placed on undertaking rural employment remains unchanged among nurses who are two- and four-years post-graduation, with little change evident when this same cohort of participants were final year and newly graduated nurses respectively [7]. We further postulate that in respect to the decisions to take up rural employment there is little change over time, or at least in the first four years after graduation. This highlights the student phase of the nurse’s journey represents a ripe time to stimulate their thinking about future employment, particularly practicing rurally. It underscores the importance that higher education providers in capitalising on the opportunities to expose students to diverse rural experiences as part of their program of study [29, 30]. In line with existing research [31, 32], the findings from the current study suggest that once a student graduates the window of opportunity to influence rural employment considerations vastly diminishes.

The participants from the current study identified they were engaged in management roles as early as their second-year post-graduation, with increasing frequency for those who are four-years post-graduation. Perhaps unsurprisingly, a sequalae of the significant rates of attrition from the nursing profession globally [33], is an increase in the reliance of more junior, less experienced nurses to lead complex care situations as well as large multidisciplinary teams [34]. This is turn has implications for staff wellbeing and ultimately on quality of patient care [34, 35]. We suggest that the widespread pressure on early carer nurses identified elsewhere [36], has implications for policymakers to leverage or mandate that health services provided management and leadership training as part of their suite of induction learning [37].

Research has suggested that the new generation of graduates– millennials– representing the future nursing workforce, have been identified as having high levels of cohesion, albeit they represent a group that is more sensitive to stress and adversity [38]. Moreover, modern ECNs have benefited from, and desired, approval from nurse leaders who have the ability to improve job satisfaction through the provision of direct training to support their adaptation to stressful situations [39]. We suggest there is a strong opportunity for policymakers and researchers to better stem the future loss of nurses from the profession as we improve our understanding and capitalise on the nuances that impact decision-making regarding employment. Given leaving employment can be mitigated, it is often an insidious indication and a precursor of leaving the profession altogether [40].

In combination, these findings support the idea that there is a period of relative stability or equilibrium that is common within the nursing workforce across the first five years post-graduation nationally [41] and globally [42]. This consistency with existing research would suggest that there is a high probability that a number of participants– specifically those that are four-years post-graduation– are going to leave the profession within the next 1 to 2 years. While any signs of an imminent departure from the nursing profession are not evident within the findings presented, we postulate there are likely to be a number of highly nuanced factors that have not been captured here that may have a role to play in the attrition of nurses as identified elsewhere [4, 5].

We suggest that the notion of a *Wicked Problem* framework provides utility for considerations of nurse retention. First coined by Rittel and Webber [43], Wicked Problems are defined as difficult to identify, open to change across time and across differing contexts, and often having several stakeholders with their own values and priorities [44]. The label of Wicked Problem for issues of retention has been used elsewhere [45] and we echo the idea that this model cogently captures the inexorable immixture of macro (institutional level), meso (ward/clinical area level) and micro (personal/individual nurse level) features at work in any considerations of nurse retention [4].

### Limitations

The low response rate raises concerns about potential report biases, which can introduce instability in the obtained results and sufficient for drawing reliable conclusions. In addition, the low response rate made it impracticable link student data across the previous five years of data collection and subsequent data matching, further impacting data analysis. This has implications for robust longitudinal understanding of change over time amongst individuals and therefore the findings presented here need to be considered cautiously. This study and future studies could be enhanced through opportunities for qualitative understandings of the meaning and experiences of the ECNs themselves. This is work that is currently being undertaken by the research team.

## Conclusion

This examination of early career nurses, are in their second and fourth-year post-graduation, suggests that there are no significant changes between the two cohorts based on self-efficacy, grit, and career aspirations. Interestingly, when we consider longitudinally there is a stability for these measures at the conclusion of their Bachelor of nursing program– finished two and four years ago respectively– amongst this cohort participants. We suggest that this period of equilibrium is in line with our current understanding of nursing retention and might be construed as the ‘calm before the storm’ which is seemingly inevitable from five years post-graduation onwards. In culmination, nursing retention is a Wicked Problem that is unavoidably a complex amalgam of macro, meso and micro factors that we are yet to full capture but are beginning to appreciate.

## Data Availability

The datasets used and/or analysed during the current study are available from the corresponding author on reasonable request.
